# Lassa Fever: Viral Replication, Disease Pathogenesis, and Host Immune Modulations

**DOI:** 10.3390/pathogens9060437

**Published:** 2020-06-03

**Authors:** Hinh Ly

**Affiliations:** Department of Veterinary Biomedical Sciences, University of Minnesota, Twin Cities, 1988 Fitch Ave., Ste. 295, Saint Paul, MN 55108, USA; hly@umn.edu; Tel.: +612-625-3358; Fax: +612-625-0204

**Keywords:** Lassa virus, arenavirus, mammarenavirus, vaccine, virulence, pathogenesis, innate immunity, adaptive immunity, COVID-19

## Abstract

Despite major discoveries made in the last few decades about Lassa fever, there are still many unresolved key issues that hamper the development of effective vaccines and therapies against this deadly disease that is endemic in several West African countries. Some of these issues include the lack of a detailed understanding of the viral and participating host factors in completing the virus life cycle, in mediating disease pathogenesis or protection from disease, and in activating or suppressing host innate and cellular immunity against virus infection, as well as of the animal models required for testing vaccines and therapeutics. This Special Issue is devoted to understanding some of these important issues and to exploring the current status of the research and development in combating Lassa fever.

Since the first case of the new coronavirus disease in 2019 (COVID-19) was reported on 25 February 2020 in Algeria (North Africa), the overall numbers of reported cases and deaths have been increasing exponentially in recent weeks in Africa, with all 47 member states of the WHO African Region being affected, and more than half of the countries in the region are experiencing community transmission (https://www.afro.who.int/health-topics/coronavirus-covid-19). As of 19 May 2020, the highest case load has been observed in West African regions at 40% of the total number of cases (24,836 cases with a case fatality rate (CFR) of 2.2%), followed by the Southern African region at 27% (17,450, CFR 1.8%), the North African region at 8.5% (7377, CFR 7.6%), the Central African region at 15% (9331, CFR 3.2%), and the East African region at 7% (4506, CFR 2.4%). A major concern is that the current COVID-19 pandemic might become endemic in some countries in Africa and around the globe. It is important to note that other infectious diseases, such as HIV, malaria, and Lassa fever, have already been endemic for many decades in Africa, in particular in Sub-Saharan Africa. It is, therefore, important not to lose sight of these ongoing pandemic and endemic infections and the potential of the co-circulation of these deadly human pathogens in these regions where health care and social and political conditions (in some countries) are highly unstable.

In this Special Issue, that is devoted to understanding some of the important issues about Lassa fever that is endemic in several West African nations, there are seven timely contributions in the form of original research and review articles on Lassa fever’s viral replication, the disease pathogenesis and protection, host immune modulations, and other related hot topics. In particular, three review articles contributed by leading researchers in the field that cover important concepts, including how different animal models can be developed for understanding Lassa fever’s disease pathogenesis and pathologies and for the evaluation of candidate vaccines and antiviral therapies [1], how to improve the breadth of host immune responses to Lassa vaccination [2], as well as how different virus and host factors orchestrate the intricate processes of Lassa virus (LASV) entry and genome replication [3]. In addition to these insightful review articles, two research articles deal with novel strategies of developing vaccines for Lassa fever, including the use of the LASV-like particles composed of the viral matrix (Z) and envelope glycoprotein complex (GPC) expressed by the modified vaccinia Ankara virus to protect mice against lethal virus challenges [4] and the use of a candidate LASV vaccine known as ML29 that is composed of the reassorted genome between the pathogenic LASV and the non-pathogenic Mopeia virus (MOPV), which shows its highly attenuated phenotype in rodents [5]. An intriguing observation from this study is that the persistent infection of cells with ML29 can result in the generation of interfering viral particles that can induce a potent level of cell-mediated immunity and can strongly interfere with the replication of arenaviruses, such as LASV, MOPV, and lymphocytic choriomeningitis virus (LCMV). 

There are two other research papers that cover some hot topics in the field. One such paper uses state-of-the-art mass spectrometry to identify novel phosphorylation sites in the LASV Z protein [6]. In particular, residues of two serines (S18, S98) and a tyrosine (Y97) located in the flexible N- and C-terminal regions of the protein are found be phosphorylated. Two of these residues, Y97 and S98, happen to be located in or directly adjacent to the so-called late domain with the PPXY motif, which is known to be required for mediating optimal progeny virion release from the infected cells. The authors showed that phosphorylation of these amino acids served as an important regulatory mechanism of virus release and that host-driven, reversible phosphorylation processes might play an important role in the regulation of LASV Z protein function in virus assembly and release.

Another important step in the life cycle of LASV is viral entry, which is a two-step process that involves the viral envelope glycoprotein complex (GPC). The GP subunit 1 (GP1) of the GPC first binds to the cell surface receptor before the viral particle is engulfed into the cellular endosome. A drop in pH in the endosome triggers GPC structural rearrangements which lead the GP subunit 2 (GP2) of the GPC to form a six-helix-bundle that helps fuse the lysosomal membrane with the LASV envelope membrane, allowing the LASV genome to enter the cellular cytoplasm for replication and transcription. In order to identify amino acid residues in GP2 that are crucial for the process of LASV entry, Willard and colleagues performed a semi-saturated alanine scanning mutagenesis of amino acid residues of GP2 [7]. They tested these mutant GPCs for efficient GP1-GP2 cleavage, cell-to-cell membrane fusion, and transduction into cells expressing the known cellular receptor (α-dystroglycan) and other secondary LASV receptors. Using this systematic experimental approach, the authors successfully identified seven GP2 mutants that could mediate efficient GP1-GP2 cleavage, yet they were unable to effectively transduce cells, which suggests that these key residues are critically important for GP2 function in the process of LASV entry into cells.
